# Secretagogin Downregulation Impairs Nerve Cell Migration in Hirschsprung Disease via Inhibition of the LEF-1/NCAM1 Axis

**DOI:** 10.1016/j.mcpro.2025.101032

**Published:** 2025-07-11

**Authors:** Yun Zhou, Shuiqing Chi, Shuai Li, Zhibin Luo, Liying Rong, Mengxin Zhang, Yunshang Chen, Jialing Guo, Dehua Yang, Xi Zhang, Guoqing Cao, Shao-tao Tang

**Affiliations:** 1 Department of Pediatric Surgery, Union Hospital, Tongji Medical College, Huazhong University of Science and Technology, Wuhan, China; 2 Hubei Key Laboratory of Precision Radiation Oncology, Wuhan, China; 3 Department of Pediatrics, The First Affiliated Hospital, Zhejiang University School of Medicine, Hangzhou, China

**Keywords:** Hirschsprung disease, ENS migration, SCGN, NCAM1

## Abstract

Hirschsprung disease (HSCR) is a common peripheral neurodevelopmental disorder and impaired enteric neural crest cell migration is one of the key factors. Secretagogin (SCGN) has been demonstrated to play a critical role in the rostral migratory stream during central nerve regeneration. However, there is a paucity of knowledge on the role of SCGN in enteric neural crest cell migration. Here, we revealed a significant downregulation of SCGN by protein profiles using tandem mass tag in HSCR lesion colon tissues. We identified decreased expression of SCGN could hinder cell migration *in vitro* and *in vivo*. Mechanistically, SCGN upregulated the transcription factor (lymphoid enhancer-binding factor 1 [LEF-1]), which directly activated the transcription of the cell adhesion molecule (neural cell adhesion molecule 1 [NCAM1]), thereby promoting cell migration. In conclusion, this study elucidates the role of SCGN in HSCR pathogenesis by demonstrating its involvement in affecting neural crest cell migration through the lymphoid enhancer-binding factor 1/neural cell adhesion molecule 1 axis. The findings could contribute to the diagnostic and therapeutic strategies for HSCR.

## Highlights

• Quantitative proteomic profiling reveals significant SCGN downregulation in HSCR patient tissues.
• SCGN deficiency impairs enteric neural crest cell migration via the LEF-1/NCAM1 axis.
• SCGN demonstrates high sensitivity and specificity for HSCR diagnosis.

## Graphical Abstract

**Figure:**
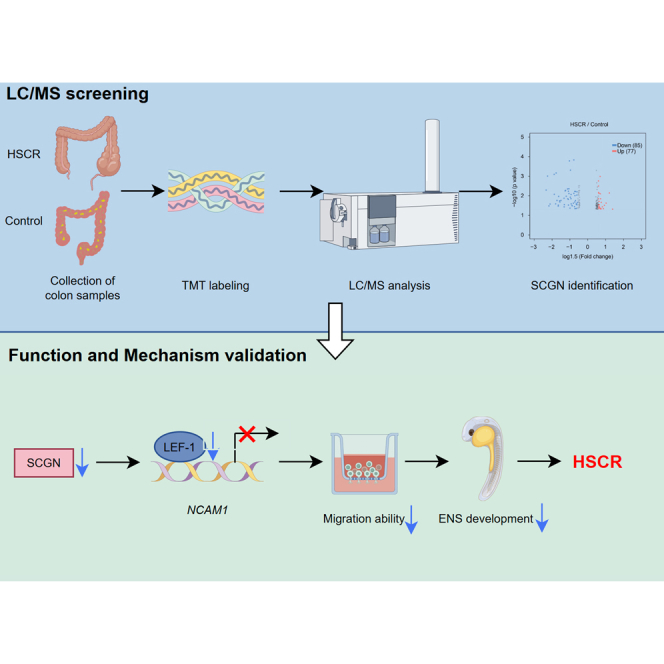

## In Brief

This study employs TMT-based proteomics to identify SCGN as a key regulator in Hirschsprung disease (HSCR). Through *in vivo* and *in vitro* analyses, we demonstrate that SCGN deficiency impairs neural crest cell migration by disrupting the LEF-1/NCAM1 axis. Notably, SCGN exhibits high sensitivity and specificity for HSCR diagnosis. Our findings reveal a novel pathogenic mechanism and position SCGN as a promising diagnostic biomarker and therapeutic target for HSCR.

Hirschsprung disease (HSCR) is a common developmental disorder of the enteric nervous system (ENS), with an incidence ranging from 1.0 to 2.6 per 10,000 live births globally (1). HSCR is characterized by the lack of ENS in the distal rectum and variable lengths of the proximal intestine. This lack of ganglion cells causes a functional blockage in the intestine, forming a narrow distal bowel and a consequent proximal distension (2). Delayed diagnosis in the neonatal period can lead to complications such as Hirschsprung-associated enterocolitis and intestinal perforation, which can be life-threatening (3). Therefore, further research into the pathogenesis of HSCR is essential for developing improved diagnostic methods and outcomes for children with this condition.

HSCR is primarily caused by the incomplete migration of vagal neural crest cells from cranial to caudal direction during development (4). During embryogenesis, enteric neural crest cells originating from the vagus nerve migrate from cephalad to caudal along the digestive tract, colonizing the intestinal wall and forming the enteric neural network. Consequently, impaired migration of these cells is one of the key factors in HSCR pathogenesis, contributing to the absence of enteric ganglion cells within the intestinal wall (5). However, the mechanisms regulating enteric neural crest cell migration remain incompletely understood.

Secretagogin (SCGN) is a calcium receptor protein with an EF-hand structure that is exclusively expressed in the neuroendocrine system, including enteric neurons and enteroendocrine cells (6). It is involved in biological processes such as cytosolic fusion, endoplasmic reticulum stress, and extracellular secretion (7). The expression of SCGN has been reported to be downregulated in neurodegenerative diseases, including Alzheimer's disease and Parkinson's disease (8). Additionally, SCGN has been associated with various neurodevelopmental disorders, such as autism and schizophrenia (9, 10), suggesting its potential role in central nervous system development. Recent studies have demonstrated that SCGN mediates MMP-2 cytosolization and activation in a membrane-associated protein V-dependent manner, which restructures the extracellular matrix and promotes the rostral migration stream (11). However, the function of SCGN in ENS migration and its potential involvement in HSCR pathogenesis remain to be investigated.

In this study, we observed low expression of SCGN in HSCR lesion colon tissues through protein profiling. Our findings indicate that SCGN plays a functional role in the migration of enteric neural crest cells and is involved in the pathogenesis of HSCR by upregulating the lymphoid enhancer-binding factor 1 (LEF-1)/neural cell adhesion molecule 1 (NCAM1) axis. Additionally, SCGN labeling for ganglion cells exhibited high sensitivity and specificity in HSCR mucosal biopsies, suggesting its potential for clinical translation.

## Experimental Procedures

### Colon Tissue Collection

This study enrolled children with constipation who first presented to our center between 2019 and 2023 and underwent partial colectomy. The children's primary complaints included severe bowel problems presenting in the neonatal period or chronic intractable constipation presenting in childhood. HSCR (International Classification of Diseases: Q43.1) and Hirschsprung allied disease (International Classification of Diseases: Q43.105) were included separately. In addition, three specimens of colon as partially resected colon due to abdominal trauma were used as normal colon specimens. Preoperative mucosal biopsy tissues and postoperative colonic tissues were stored. General information about the children, including gender, ethnicity, age at presentation, and treatment experience, was carefully recorded. All experimental protocols involving human participants were reviewed and approved by the Ethics Committee Office of Union Hospital, Tongji Medical College, Huazhong University of Science and Technology, with the approval number 2016[S180]. For human studies, all procedures were conducted in accordance with the ethical principles outlined in the Declaration of Helsinki, including obtaining informed consent from participants or their legal guardians where applicable.

### Human Tissue Preparation

Postoperative colon specimens from HSCR patients were classified into narrow, transition, and distended segments based on intestinal histomorphology. All collected tissues including HSCR group and the control group were divided into three parts: one part was snap-frozen in liquid nitrogen and stored at −80 °C for proteomics analysis; another part was immersed in RNAlater (Invitrogen, catalog no. AM7021) for 24 h and then stored at −80 °C for RNA extraction; and the remaining part was soaked in formalin solution and embedded in paraffin for immunohistochemical staining.

### TMT Protein Profile Sequencing

Colon tissue specimens from three HSCR and three trauma cases were utilized for sequencing. The samples were pulverized into fine powder using a mortar and pestle under liquid nitrogen conditions. Subsequently, five volumes of trichloroacetic acid and acetone solution (volume ratio 1:9) were added, followed by precipitation at −20 °C for over 4 h. The supernatant was centrifuged at 6000*g* at 4 °C for 40 min, and the supernatant was discarded. Precooled acetone was added, and the samples were washed three times. The precipitate was dried in a fume hood. Twenty to thirty milligrams of dried powder were weighed and resuspended in 30 times the volume (m/v) of SDT lysate using a vortex mixer. The mixture was then incubated in boiling water for 5 min. Ultrasonic disruption of the powder was followed by another incubation in boiling water for 15 min. Centrifugation at 14000*g* for 15 min was performed, and the supernatant was filtered through a 0.22 μm filter membrane. The filtrate was collected and divided into aliquots for storage at −20 °C.

For each sample, 150 μg of protein solution was taken, reduced with 5 mM DTT for 30 min at 56 °C, and then alkylated with 15 mM iodoacetamide for 30 min at room temperature in the dark. The reaction was quenched with 30 mM cysteine for an additional 30 min at room temperature. The peptides were desalted using a C18 cartridge, lyophilized in 40 μl of 0.1% formic acid solution, and quantified by measuring the absorbance (A) at 280 nm (A280).

Each sample was separated using a nanoliter flow rate Easy nLC system. Buffer A consisted of 0.1% formic acid in water, and buffer B consisted of 0.1% formic acid in acetonitrile (80% acetonitrile). The column was initially equilibrated with 100% buffer A, and the sample was loaded by an autosampler onto an analytical column (Thermo Fisher Scientific, Acclaim PepMap RSLC 50 um × 15 cm, nano viper, P/N164943) at a flow rate of 300 nl/min. A gradient of 1.5 h was then applied: 0 to 5 min, 6% buffer B; 5 to 75 min, linear gradient from 6% to 38% buffer B; 75 to 85 min, linear gradient from 38% to 100% buffer B; 85 to 90 min, maintained at 100% buffer B. The samples were separated by chromatography and analyzed by mass spectrometry using a Q Exactive mass spectrometer. The analysis time was 60 min in positive ion detection mode. The scanning range of the parent ion was 350 to 1800 *m/z*, the primary mass spectrum resolution was 70,000, the automatic gain control target was 3e6, and the maximum IT of the primary mass spectrum was 50 ms. Peptide and peptide fragment *m/z* were collected according to the following method: 10 fragments were collected after each full scan (mass spectrometry 2 [MS2] scan). The MS2 activation type was HCD, the Isolation window was 2 *m/z*, the secondary mass spectra resolution was 35,000, the microscans was 1, the maximum IT of the secondary mass spectra was 45 ms, and the normalized collision energy was 30 eV. Quantitative proteomics analysis for tandem mass tag (TMT) was performed using a Q Exactive high-resolution mass spectrometer. MS2 maps were obtained using the fast HCD mode. Additional search parameters and acceptance criteria for peptide/protein identification were provided in the Supplemental Table S6.

### Cell Culture

The cell lines used in the study (SH-SY5Y and SK-N-BE(2)) were obtained from the American Type Culture Collection, to emulate in neuroblastoma cells with different degrees of differentiation. The cells were dissociated using 0.05% EDTA-trypsin, and cultured in minimum essential medium (MEM) medium supplemented with 10% fetal bovine serum, 100 units/ml penicillin, and 100 μg/ml streptomycin. The cultures were maintained at 37 °C in an atmosphere of 5% CO2. The cell lines were authenticated by short tandem repeat profiling and regularly tested for *mycoplasma* contamination.

### RNA Interference

The siRNA and Lipofectamine RNAiMAX were mixed thoroughly with appropriate volumes of Opti-MEM I serum-free medium, respectively. After standing at room temperature for 5 min, the two mixtures were gently combined and incubated at room temperature for an additional 20 min. The mixture was then added to cells at a density of 30 to 40% and placed in a constant temperature incubator at 37 °C for 48 h of incubation. The siRNA sequences used in the study were shown in Supplemental Table S1.

### Plasmid Transfection

The plasmid and Lipofectamine 2000 were mixed with an appropriate volume of Opti-MEM I serum-free medium and incubated for 5 min. The two tubes were then gently mixed and left at room temperature for 20 min. The mixture was added to cells at 70 to 90% confluence, and the cells were placed in a 37 °C incubator for 24 h of incubation.

### Antibodies and Reagents

Primary antibodies and reagents used in this study included rabbit anti-SCGN (Proteintech, 13606-1-AP, 1:2000), mouse anti-GAPDH (Proteintech, 60004-1-Ig, 1:1000), rabbit anti-LEF-1 (Abcam, ab137872, 1:2000), rabbit anti-NCAM1 (Proteintech, 60238-1-AP, 1:2000), mouse anti-Flag (Abclonal, AE005, 1:2000), and polymeric immunoglobulin receptor ([PIGR] Abcam, ab275020, 1:1000).

### Western Blot Analysis

Colon tissue was ground on ice to form a tissue suspension, followed by digestion into a cell suspension using a protease mixture. Cells derived from tissues or cultures were collected into centrifuge tubes, and proteins were extracted using NETN lysis solution (20 mM Tris–HCl (pH 8.0), 100 mM NaCl, 1 mM EDTA, and 0.5% Nonidet P-40) with an appropriate amount of 5x SDS, and heated at 100 °C for 10 min. Following SDS-PAGE electrophoresis, membrane transfer, blocking, primary antibody and secondary antibody incubation, proteins were detected using a chemiluminescence instrument (UVP ChemiDoc-It). After SDS-PAGE electrophoresis, membrane transfer, blocking, primary antibody incubation, and secondary antibody incubation, the expression levels of target proteins were detected using a chemiluminescence instrument (UVP ChemiDoc-It 510).

### Real-Time qPCR

Total cellular RNA was extracted according to the instructions of the RNA extraction kit (Omega), and after reverse transcription of RNA into the complementary DNA using HiScript III-RT SuperMix, RT-qPCR reactions were performed using ChamQ SYBR qPCR Master Mix. The primer sequences are shown in Supplemental Table S2.

### Immunohistochemical Staining

Colon and mucosal tissues were fixed by formalin immersion for 12 to 24 h, followed by dehydration in a gradient ethanol solution, clearing with xylene, and embedding in paraffin. The paraffin-embedded tissues were sectioned, subjected to EDTA antigen retrieval in a water bath at 100 °C, quenched for endogenous peroxidase activity with H_2_O_2_, and blocked with 5% BSA solution at 37 °C. After incubation with the appropriate primary and secondary antibodies, the sealed tissue sections were stained with 3,3'-diaminobenzidine dye and hematoxylin, and mounted with resin. Each biopsy image was individually scored by three pathologists.

### CCK-8 AssayCCK-8 Assay

Cells were cultured at a density of 2000 cells per well in 96-well plates. Cell Counting Kit 8 (CCK8) solution was added to each well, and absorbance values at 450 nm were measured every 12 h for a total of 72 h using a spectrophotometer (EnSpire 2300).

### Transwell Assay

Cells were seeded into 24-well plates containing an appropriate volume of complete medium. Following trypsinization and dilution with serum-free medium, 5 × 10^4^ cells were added to each chamber and incubated at a constant temperature of 37 °C. After a 24-h incubation period, the chambers were fixed with 4% paraformaldehyde (PFA) and stained with crystal violet solution. The excess staining solution was removed by washing with water, and the number of cells that had penetrated the chambers in each group was subsequently observed and photographed under a microscope.

### Scratch Healing Assay

Cells were seeded into 6-well plates and cultured until they reached 100% confluence. Uniform-thickness scratches were then created in each well using a pipette, and the serum-free medium was replaced. The scratches were observed under a microscope (Olympus) at 0 h and 48 h, respectively, and photographs were taken to record and calculate the healing rate of the scratches in each group using ImageJ software (https://imagej.net/ij/).

### Dual Luciferase Reporter

WT and mutant plasmids were constructed based on the sequence of the NCAM1 promoter region. Cells were seeded into 24-well plates and transfected with plasmids at a ratio of Firefly:Renilla:Transfection Reagent = 0.1 μg:0.01 μg:250 μl per well. Cells were transfected with either a LEF-1 overexpression plasmid or a control plasmid, according to the experimental groups. Luciferase activity was measured using the Dual-Luciferase Reporter Gene Assay Kit (Bicentennial, RG029S) 24 h posttransfection. Subsequently, the same cells were transfected with either a LEF-1 overexpression plasmid or a control plasmid, following the respective group assignments.

### Animals

All zebrafish (*Danio rerio*) experiments were performed according to standard procedures, and both adult fish and embryos were raised at 28.5 °C in the Aquatic Ecosystems. The following lines were used in this study: AB strain (WT) zebrafish. All experimental protocols were approved by the Animal Ethical Committee, Wuhan Union Hospital of Huazhong University.

### Morpholino and mRNA Injections

The sequence of MO and control is detailed in Supplemental Table S3. Morpholino oligonucleotide MO1 targets the translational initiation site of the zebrafish SCGN gene, thereby inhibiting protein synthesis, while MO2 is designed to disrupt normal gene splicing processes. A standard mismatch morpholino was employed as a negative control. Microinjections were performed at the single-cell developmental stage, with administration into both the yolk and cytoplasm. The standard injection parameters consisted of 5 ng morpholino and/or 200 pg mRNA per injection, except where specifically noted for experimental variations.

### *In Situ* Hybridization

At 72 h postfertilization, the specimens were preserved and treated with proteinase K (1 mg/ml) for 20 min at ambient temperature, followed by secondary fixation using 4% PFA.

Subsequently, the samples were exposed to digoxigenin-tagged SCGN antisense RNA probes. Following probe removal, they were treated with an alkaline phosphatase–linked anti-digoxigenin antibody (diluted 1:2000) and stored overnight at 4 °C. After thorough rinsing, the samples underwent chromogenic detection using nitroblue tetrazolium and 5-bromo-4-chloro-3-indolyl-phosphate substrate ([NBT/BCIP], Roche) following the supplier’s protocol.

### Immunofluorescence

Zebrafish larvae (3 days post-fertilization) were immersion-fixed in 4% PFA prepared in PBS (pH 7.4) for 3 h at ambient temperature or alternatively at 4 °C overnight. Following fixation, specimens were washed extensively with PBS, equilibrated in 30% sucrose for cryoprotection, and subsequently embedded in optimal cutting temperature (Tissuetek) prior to cryosectioning at 10 μm.

Tissue sections were subjected to overnight incubation with primary antibodies (1:200, Molecular Probes) prepared in PBS containing 0.2% Triton X-100. After thorough PBS washes, sections were exposed for 2 h to species-matched secondary antibodies conjugated with Alexa Fluor 488 (1:500, Molecular Probes), similarly diluted in 0.2% Triton X-100/PBS. Finally, cellular nuclei were visualized by counterstaining with 4′,6-diamidino-2-phenylindole following standard protocols.

### Experimental Design and Statistical Rationale

In this study, the sample sizes for each experiment were determined based on preliminary data, established protocols in the field, and statistical power considerations. For the TMT-based proteomic screening, we analyzed 6 human tissue samples (3 HSCR and 3 controls), a sample size consistent with discovery-phase proteomic studies, which balances the trade-off between detecting meaningful biological variations and minimizing false positives. The sample size for clinical validation (n = 130, 52 HSCR *versus* 78 controls) was determined based on a power analysis for receiver operating characteristic curve analysis (expected area under the curve ≥0.8, α = 0.05, power = 90%). This exceeds the minimum requirement (n = 70) calculated using the method of Hanley and McNeil (1982), ensuring robust evaluation of SCGN's diagnostic performance.

The TMT sequencing portion utilized Proteome Discoverer 2.1 (Thermo Fisher Scientific, https://www.thermofisher.cn/cn/zh/home/industrial/mass-spectrometry/liquid-chromatography-mass-spectrometry-lc-ms/lc-ms-software/multi-omics-data-analysis/proteome-discoverer-software.html) software to convert the raw map files (.raw files) generated by the Q Exactive into .mgf files. These .mgf files were subsequently submitted to the MASCOT 2.6 server for database searching using the software's integrated tool. The database search file (.dat file) generated by the MASCOT server was then transferred back to Proteome Discoverer 2.1, and the data were filtered according to a false discovery rate threshold of less than 0.01.

For the real-time quantitative PCR (qRT-PCR), cell scratch, and protein gel electrophoresis data, a *t* test was employed to analyze statistical differences between two groups of independent samples. The ANOVA was used to assess statistical differences among multiple groups of independent samples. The chi-square test was applied to analyze differences in SCGN expression within the colonic mucosal tissues of individuals with HSCR and normal controls. Statistical differences were considered significant at *p* < 0.05 (n.s. no statistical difference, ∗*p* < 0.05, ∗∗*p* < 0.01, and ∗∗∗*p* < 0.001).

## Results

### SCGN is Lowly Expressed in HSCR Lesion Colon Tissue

**Fig. 1:**
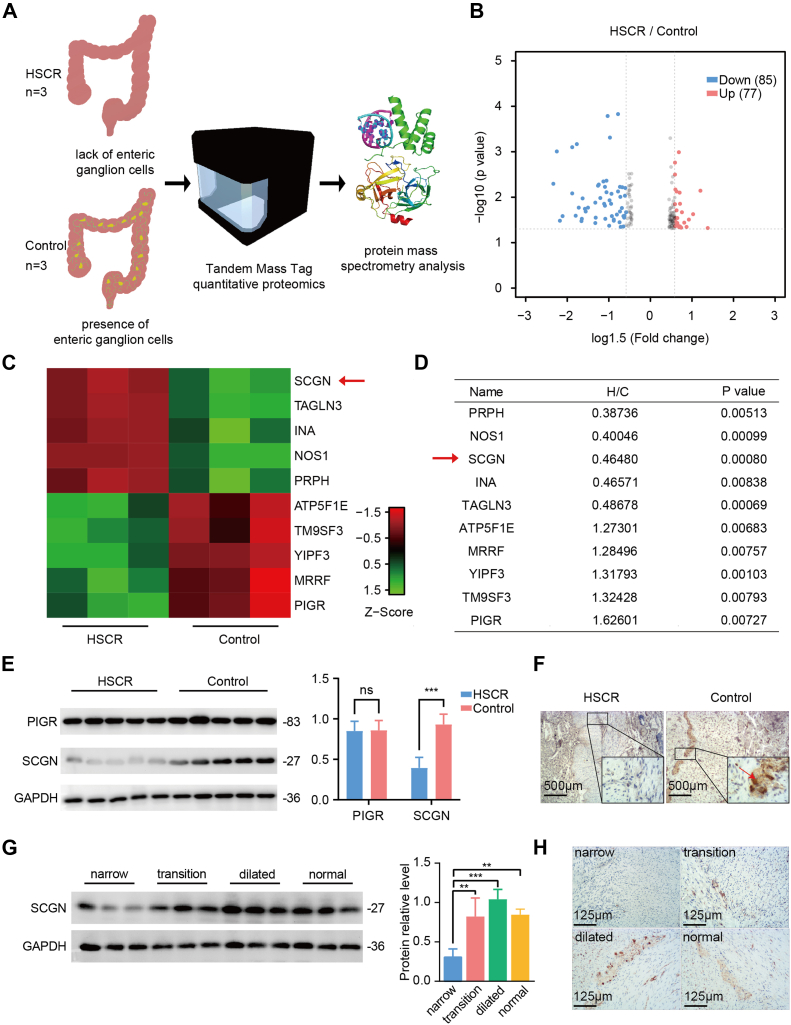
**SCGN exhibits low expression in HSCR lesion colon tissue.***A*, colonic tissues of diseased segments from 3 HSCR patients and 3 controls (trauma) were detected by TMT protein profiling. *B*, differential gene volcano plot. Proteins with an absolute fold change of 2 or greater were considered differentially expressed. A total of 162 proteins were identified as differentially expressed: 77 were upregulated in HSCR (*red dots*), while 85 were downregulated in HSCR (*blue dots*). *C*, TMT protein profiling sequencing of the top 10 proteins clustering by fold difference sorted top 10 proteins clustering heatmap. *D*, list of names, fold change values and *p* values of top 10 proteins sorted by fold difference. *E*, *left panel*: protein levels of SCGN and PIGR in control and HSCR children's colon tissues were detected by Western blot; *right panel:* the *gray values* of the bands were measured by using ImageJ software, and the relative protein expression of SCGN and PIGR with respect to β-actin was calculated, and the data were expressed as means ± SDs. ∗∗∗*p* < 0.001 (n = 5). *F*, SCGN expression in control and HSCR colon tissues was labeled by immunohistochemical staining; the location of the enlarged image is at the junction of the circular and longitudinal muscles of the intestinal wall, and the *red arrows* are SCGN^+^ ganglia. *G*, levels of SCGN protein expression in the narrow, transition, and dilated segments of HSCR and the nonconstipation colons were detected by WB. *H*, intermuscular SCGN expression in the intestinal wall was labeled by immunohistochemical staining, and was negative in narrow segments, positive in the transition segment, the dilated segment, and the nonconstipation colons. HSCR, Hirschsprung disease; SCGN, secretagogin.

To identify key molecules involved in the development of intestinal neural crest cells, colon tissues from patients with HSCR were compared with those from individuals with abdominal trauma. Differential protein analysis was conducted using TMT protein profiling on three cases of diseased colon tissue from each group (Supplemental Tables S4 and S5). A total of 162 differential proteins were identified between HSCR and the control group (fold change >1.2, *p* < 0.05), with 77 upregulated and 85 downregulated (Fig. 1, *A* and *B*). The top five proteins were clustered and analyzed based on their upregulated and downregulated degrees, with the results sorted by fold change value in Figure 1, *C* and *D*. Among these, PRPH and NOS1, the most significantly downregulated proteins, have been reported to be ineffective as markers, limiting their clinical translation. The diagnostic value of SCGN (ranked third in the downregulated group) and PIGR (ranked first in the upregulated group) in HSCR remains unclear. Consequently, SCGN and PIGR were selected for further investigation. To validate the sequencing results, 10 cases of colon tissue from partial colectomy for constipation were collected, including five each from HSCR and nonHSCR patients. The expression levels of SCGN and PIGR were detected by Western blot (WB). While the expression level of PIGR in HSCR did not show significant changes compared to the control group, the expression level of SCGN in HSCR was significantly lower than the control (Fig. 1*E*). Further, the control group exhibited intense immunostaining for SCGN in the ganglia, whereas the immunoexpression in the narrow colon tissues was negative (Fig. 1*F*). Furthermore, the narrow segments devoid of enteric ganglia exhibited the lowest SCGN expression, while the transition segments demonstrated the second lowest SCGN expression. No significant difference in the level of SCGN was observed between the distended segments and normal tissues (Fig. 1, *H* and *I*). It indicated a positive correlation between SCGN expression and the number of intestinal ganglia. In conclusion, the expression of SCGN in colonic tissues from HSCR lesions was significantly reduced, suggesting its potential role in the development of enteric neural crest cells.

### SCGN Deficiency Inhibits Neuronal Cell Migration

**Fig. 2:**
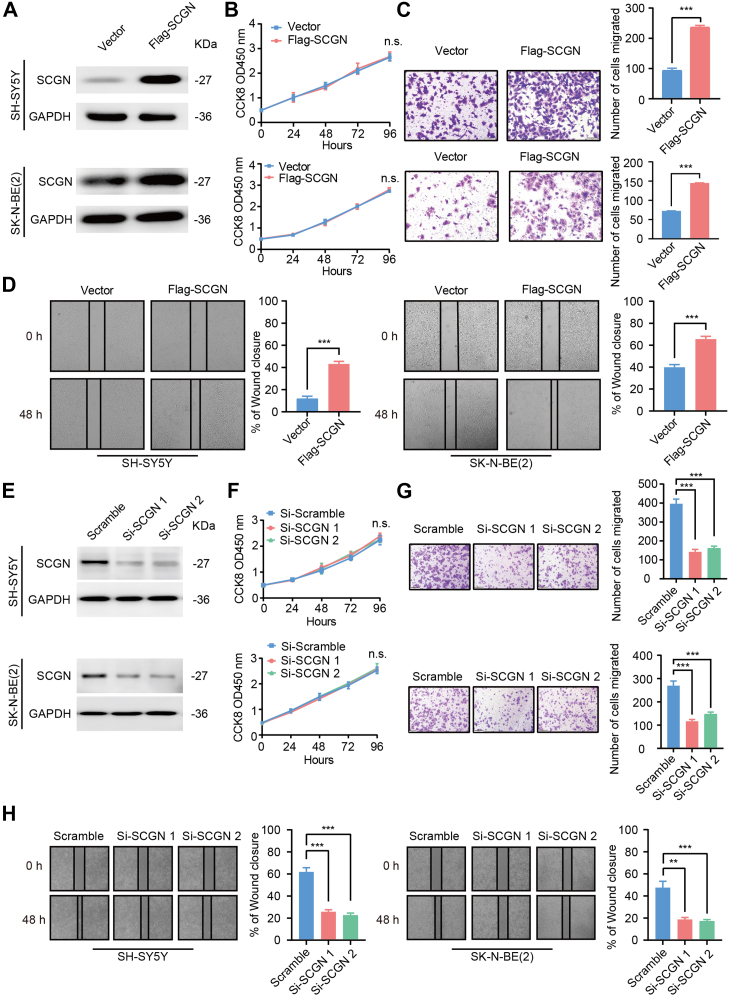
**SCGN deficiency inhibits neuroblast cell migration.***A*, SH-SY5Y and SK-N-BE(2) cells were transfected with the control vector and SCGN vector, respectively, and the expression levels of SCGN proteins in the control and SCGN overexpression groups were detected by Western blot. *B*, in the CCK8 proliferation assay, there was no significant difference in the proliferation rate of cells in the SCGN overexpression group compared with that of the control group. n.s. *p* > 0.05 (n = 3). *C*, control and SCGN overexpression groups were incubated for 24 h in chambers, and the cells that passed through the chambers were fixed and stained, and the number of cells that passed through the chambers was significantly increased in the SCGN overexpression group compared with the control group. Compared with the control group, the number of cells crossing the chambers was significantly higher in the SCGN overexpression group. ∗∗∗*p* < 0.001 (n = 3). *D*, in the scratch assay, the SCGN overexpression group showed faster wound healing than the control group. ∗∗∗∗*p* < 0.0001 (n = 3). *E*, control siRNA was transfected with Si-SCGN 1 and Si-SCGN 2 in SH-SY5Y and SK-N-BE(2) cells, respectively, and the SCGN protein of the three groups was detected by Western blot expression level. *F*, the proliferation ability of cells among the control, Si-SCGN 1, and Si-SCGN 2 groups was detected by CCK-8 assay. n.s. *p* > 0.05 (n = 3). *G*, the migration ability of control, Si-SCGN 1 and Si-SCGN 2 cells in all groups was detected by transwell migration assay. ∗∗∗*p* < 0.001 (n = 3). *H*, the migration ability of control, Si-SCGN 1, and Si-SCGN 2 cells was further detected by scratch assay. ∗∗∗*p* < 0.001 (n = 3). SCGN, secretagogin.

The human neuronal cell lines SH-SY5Y and SK-N-BE(2) are frequently employed as models for studying the functional properties of enteric neural crest cells, particularly in the context of HSCR. To elucidate the biological role of SCGN in the development of intestinal neural crest cells, we investigated the proliferation and migration capacities of these cell lines following manipulation of SCGN expression. We transfected the control plasmid and Flag-SCGN plasmid into SH-SY5Y and SK-N-BE(2) cells. After transfection of Flag-SCGN, the level of SCGN protein significantly increased (Fig. 2*A*). However, there was no significant difference in the proliferation rates of cells between the two groups, as determined by CCK8 assays (Fig. 2*B*). Transwell assays revealed a significantly higher number of cells migrating through the cellular compartment in the SCGN-overexpressing group than the control group, suggesting enhanced migration ability (Fig. 2*C*). This finding was corroborated by the faster scratch healing observed in SH-SY5Y and SK-N-BE(2) cells overexpressing SCGN (Fig. 2*D*). To validate these findings, we conducted functional experiments after silencing SCGN expression using siRNA. We observed that SCGN knockdown significantly impaired the migration ability of SH-SY5Y and SK-N-BE(2) cells while having no effect on their proliferation rate (Fig. 2*E*-*H*). In summary, our results demonstrate that SCGN promotes the migration of neuronal cells without affecting their proliferation ability.

### SCGN Controls Zebrafish Neuronal Development

**Fig. 3:**
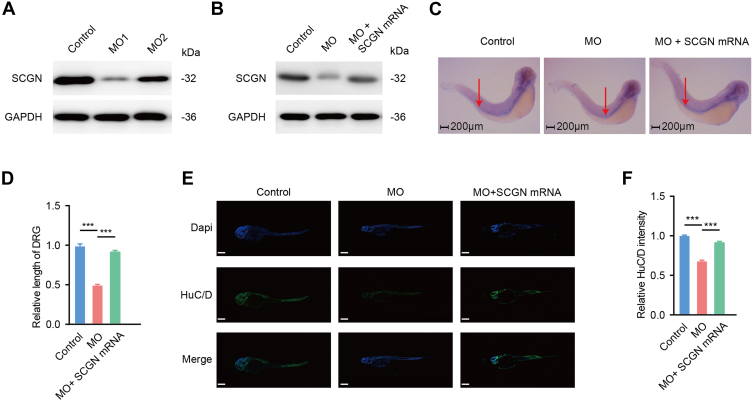
**SCGN controls zebrafish neuronal development.** MO1: MO1 sequence-injected knockdown group; MO2: MO2 sequence-injected knockdown group; MO+SCGN mRNA: coinjection group of MO1 sequence and SCGN mRNA plasmid. All injections were performed at the one-cell stage of embryonic development. *A*, SCGN protein expression levels in zebrafish tissues at 72 hpf. *B*, MO1 was selected as the knockdown sequence, and SCGN expression levels were examined in the control group, MO group, and MO+SCGN mRNA coinjection group in zebrafish tissues at 72 hpf. *C*, whole-mount *in situ* hybridization staining of SCGN in zebrafish embryos at 72 hpf (10× magnification), *purple* indicates the zebrafish spinal nerves, while *red arrows* denote the nerve terminals. *D*, relative length of the median rhombic nerve in zebrafish embryos (mean ± SD, n = 5, ∗∗∗*p* < 0.001). *E*, immunofluorescence staining of HuC/D in zebrafish embryos at 72 hpf (10× magnification). *F*, quantitative analysis of relative green fluorescence intensity in zebrafish embryos (mean ± SD, n = 5, ∗∗∗*p* < 0.001). hfp, hour postfertilization; SCGN, secretagogin.

To further investigate the biological role of SCGN *in vivo*, we selected zebrafish (*D. rerio*), a widely utilized model organism for neurodevelopmental studies, as our research subject. The zebrafish SCGN sequence exhibits high homology with its human counterpart. To suppress SCGN expression, we designed two distinct morpholino sequences for knockdown experiments in zebrafish embryos, thereby mitigating potential off-target effects. In 3-day-old zebrafish larvae, we examined SCGN expression patterns across tissues. Both morpholinos effectively inhibited SCGN expression (Fig. 3*A*), with morpholino 1 demonstrating superior knockdown efficiency. Consequently, MO 1 was selected for all subsequent experiments. We coinjected MO with SCGN mRNA into zebrafish embryos. Western blotting analysis revealed a partial restoration of SCGN protein expression levels compared to MO-only controls (Fig. 3*B*). Through *in situ* hybridization, we mapped the spatial distribution of SCGN expression (dark purple staining, Fig. 3*C*) in zebrafish. Quantitative analysis revealed significantly shorter spinal nerves in the SCGN knockdown group than controls. Notably, this inhibitory phenotype was rescued upon supplementation with SCGN mRNA (Fig. 3*D*), indicating that SCGN knockdown impairs spinal nerve development in zebrafish. Furthermore, immunofluorescence labeling with HuC/D, a marker for mature neurons, more strikingly demonstrated that SCGN downregulation disrupts spinal nerve development (Fig. 3, *E* and *F*). This developmental impairment was effectively reversed by exogenous SCGN supplementation, confirming the essential role of SCGN in zebrafish neurogenesis.

### Cell Adhesion Molecule NCAM1 is a Downstream Molecule of SCGN

**Fig. 4:**
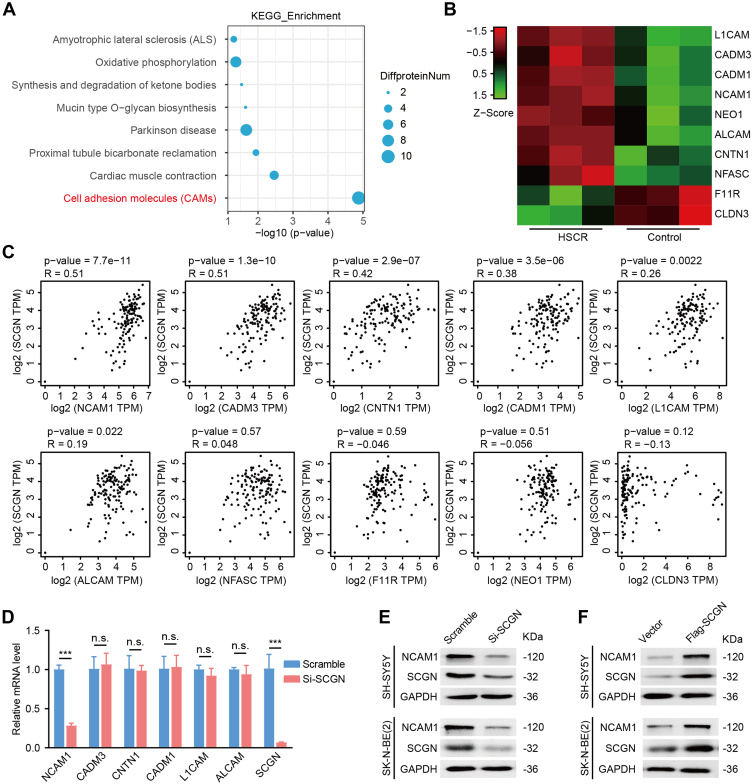
**Cell adhesion molecule NCAM1 is a downstream molecule of SCGN.***A*, Figure 1 Protein profiling data were analyzed according to KEGG pathway enrichment and plotted as bubble plots. *B*, heat maps of 10 proteins in the cell adhesion molecule pathway are shown. *C*, correlation analysis of SCGN with 10 genes in the GEPIA database, in which *NCAM1*, *CADM1*, *CNTN1*, *L1CAM*, and *CADM3* were correlated with SCGN expression. *D*, the mRNA levels of the above 5 genes were significantly decreased by qRT-PCR to detect the mRNA levels of the above five genes in Si-SCGN *cell lines*, in which the mRNA level of NCAM1 was significantly decreased, and the transcript levels of the other four genes were not significantly changed. ∗∗∗*p* < 0.001 (n = 3). *E*, protein levels of NCAM1 were detected by Western blot in Si-SCGN and control. *F*, protein levels of NCAM1 were detected by Western blot in overexpression of SCGN and control. NCAM, neural cell adhesion molecule; KEGG, Kyoto Encyclopedia of Genes and Genomes; qRT-PCR, real-time quantitative PCR; SCGN, secretagogin.

To elucidate the specific mechanism through which SCGN exerts a promigratory effect, we performed Kyoto Encyclopedia of Genes and Genomes pathway enrichment analysis on the differentially expressed genes identified in the protein profile depicted in Figure 1. This analysis revealed that the cell adhesion molecule pathway, which is intimately linked to cell migration, was the most significantly enriched (Fig. 4*A*). The clustering heatmap of genes within this pathway is presented in Figure 4*B*, illustrating their expression trends relative to SCGN. We subsequently used the GEPIA database to predict the expression correlation between SCGN and the molecules shown in Figure 4*B* in human colon tissues. Our analysis identified significant positive correlations between NCAM1, CADM1, CNTN1, L1CAM, CADM3, and SCGN (Fig. 4*C*). To assess the impact of SCGN on these molecules, we conducted qRT-PCR experiments. The results indicated a significant reduction in NCAM1 expression in SCGN-silenced cells, while the expression of the other four molecules remained unchanged (Fig. 4*D*). WB analysis further confirmed the upregulation of NCAM1 protein expression by SCGN (Fig. 4, *E* and *F*). These findings collectively suggest that NCAM1 may be a pivotal downstream substrate for SCGN in promoting the migration of neuronal cells.

### SCGN Promotes Neuronal Cells Migration by Upregulating NCAM1

**Fig. 5:**
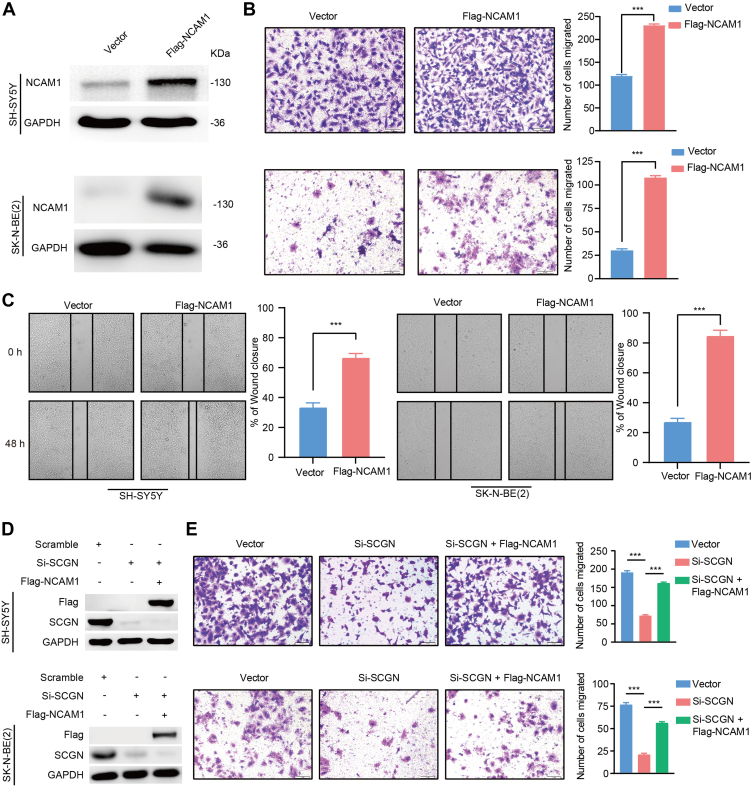
**SCGN promotes neuroblast migration by upregulating NCAM1.***A*, the control vector and NCAM1 vector were transfected in SH-SY5Y and SK-N-BE(2) cells, respectively, and NCAM1 protein expression level was detected by Western blot assay after 24 h. *B*, migration ability of the control group *versus* NCAM1 overexpression was detected by transwell migration assay. ∗∗∗*p* < 0.001 (n = 3). *C*, in the scratch assay, wound healing was accelerated in the NCAM1 overexpression group compared with the control group. ∗∗∗*p* < 0.001 (n = 3). *D*, the corresponding SiRNA and plasmid were transfected in SH-SY5Y and SK-N-BE(2) cells, respectively, and the protein content of the corresponding molecules was detected by Western blot after the cells were harvested. *E*, the migration ability of the cells in each group was detected by transwell assay. ∗∗∗*p* < 0.001 (n = 3). NCAM, neural cell adhesion molecule; SCGN, secretagogin.

NCAM1 is a neural adhesion molecule belonging to the immunoglobulin superfamily (12). It plays a critical role in neurogenesis, synapse formation, proliferation, and cell migration. Our results demonstrated that overexpression of NCAM1 increased the number of cells penetrating the chambers in SH-SY5Y and SK-N-BE(2) and accelerated the rate of cellular scratch healing, suggesting that NCAM1 promotes SH-SY5Y and SK-N-BE(2) cell migration (Fig. 5, *A*–*C*). To investigate whether NCAM1 mediates the SCGN-induced enhancement of cell migration ability, we overexpressed NCAM1 in SCGN-knockdown SH-SY5Y and SK-N-BE(2) cells and performed functional assays. Both transwell and scratch assays revealed that the reduced migratory capacity of SH-SY5Y and SK-N-BE(2) cells caused by SCGN knockdown could be restored by exogenous overexpression of NCAM1 (Fig. 5, *D*–*F*), suggesting that SCGN promotes SH-SY5Y and SK-N-BE(2) cell migration through the upregulation of NCAM1 expression.

### SCGN Directly Induces NCAM1 Transcriptional Activation by Upregulating the Transcription Factor LEF-1

**Fig. 6:**
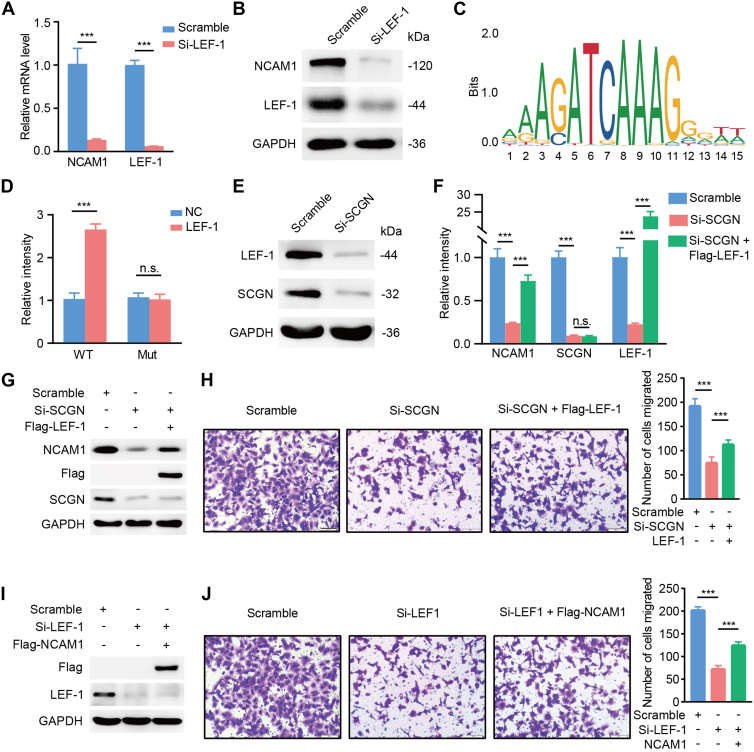
**SCGN directly induces NCAM1 transcriptional activation by upregulating the transcription factor LEF-1.***A*, NCAM1 transcript levels change with LEF-1 knockdown. *B*, NCAM1 translation levels change at the age of LEF-1 knockdown. *C*, LEF-1 and NCAM1 gene promoter binding sites were predicted by the JASPAR database. *D*, in the dual luciferase assay, the fluorescence level of the WT NCAM1 promoter group was significantly elevated after overexpression of LEF-1, and the fluorescence level of the mutant NCAM1 promoter group did not show any significant change compared with the control group. ∗∗∗*p* < 0.001 (n = 3). *E*, protein levels of LEF-1 and NCAM1 were detected by Western blot in control and SCGN knockdown groups. *F*, mRNA levels of SCGN, LEF-1, and NCAM1 were detected by qRT-PCR in the subgroups above. ∗∗∗*p* < 0.001 (n = 3). *G*, transfection of Si-SCGN in neuroblast, as well as exogenous overexpression of LEF-1, and protein expression levels of the corresponding molecules were detected by Western blot after collection of cells from each group. *H*, number of cells crossing the chambers in control, SCGN knockdown, and rep group cells in transwell experiments. ∗∗∗*p* < 0.001, n.s. *p* > 0.05 (n = 3). *I*, neuroblasts were transfected with the corresponding SiRNA and plasmid, respectively, and the protein levels of the corresponding molecules were detected by Western blot after cell collection. *J*, the number of cells crossing the chambers in control, LEF-1 knockdown, and rep group cells in transwell experiments. ∗∗∗*p* < 0.001, n.s. *p* > 0.05 (n = 3). LEF-1, lymphoid enhancer-binding factor 1; NCAM, neural cell adhesion molecule; SCGN, secretagogin.

To investigate how SCGN regulates NCAM1 expression, we hypothesized that SCGN influences upstream transcription factors of NCAM1, given its ability to upregulate NCAM1 at both transcriptional and translational levels. Previous studies have shown that LEF-1, a transcription factor, increases NCAM1 promoter activity in primary mammary stromal cells, suggesting a transcriptional regulatory relationship (13). To confirm this, we first observed that silencing LEF-1 led to the downregulation of NCAM1 expression at both mRNA and protein levels, as determined by qRT-PCR and WB analysis (Fig. 6, *A* and *B*). Subsequently, we identified a potential LEF-1 binding site within the NCAM1 promoter region using the JASPAR database (Fig. 6*C*) and constructed luciferase reporter plasmids containing either the WT NCAM1 promoter or the predicted site mutant. Luciferase assays revealed that LEF-1 overexpression significantly increased luciferase expression from the NCAM1 WT reporter plasmid but had no effect on the mutant reporter, indicating direct binding of LEF-1 to the NCAM1 promoter and its subsequent transcriptional regulation (Fig. 6*D*). Importantly, we found that LEF-1 was positively regulated by SCGN (Fig. 6*E*). Moreover, the downregulation of NCAM1 expression caused by SCGN silencing could be reversed by overexpressing LEF-1 (Fig. 6, *F* and *G*), suggesting a LEF-1–dependent mechanism for SCGN-mediated NCAM1 upregulation. Furthermore, overexpression of LEF-1 could counteract the decreased migratory ability of SH-SY5Y and SK-N-BE(2) cells induced by SCGN knockdown (Fig. 6*H*). Additionally, the inhibition of SH-SY5Y and SK-N-BE(2) cell migration caused by LEF-1 deletion was also reversed by NCAM1 overexpression (Fig. 6, *I* and *J*), further supporting the role of NCAM1 in neuronal cells migration. In conclusion, our findings demonstrate that SCGN induces transcriptional activation of NCAM1 by upregulating the transcription factor LEF-1, which subsequently enhances the migratory capacity of neuronal cells.

### SCGN Applied in Mucosal Biopsy has High Sensitivity and Specificity in HSCR Diagnosis

**Fig. 7:**
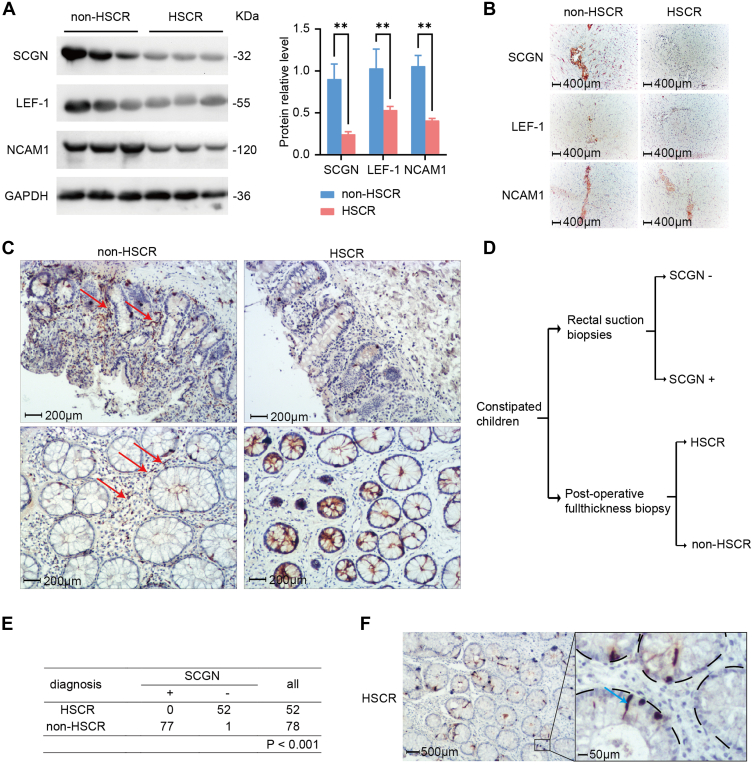
**SCGN applied in mucosal biopsy has high sensitivity and specificity in HSCR diagnosis.***A*, expression levels of SCGN, LEF-1, and NCAM1 were examined in colon tissue specimens from three patients with Hirschsprung disease (HSCR) and three non-HSCR controls. *B*, immunohistochemical staining of the same specimens was performed to localize SCGN, LEF-1, and NCAM1. The images depict the myenteric ganglion region, showing cytoplasmic expression of SCGN and NCAM1, and nuclear localization of LEF-1. In non-HSCR tissues, robust positive staining was observed for all three markers. In contrast, HSCR tissues exhibited negative staining for SCGN and LEF-1, while NCAM1 showed weak positivity. *C*, staining of SCGN in two orientated sections (the *top* is perpendicular to the intestinal wall, and the *bottom* is parallel to the intestinal wall) of HSCR and control colonic tissues. *D*, the clinical study included children with constipation and surgical treatment at our institution for mucosal biopsy. Postoperative specimens were pathologically typed. *E*, the diagnosis was confirmed by a prospective study of 130 patients who underwent mucosal biopsy and whole colon biopsy after colon resection (52 HSCR, 78 non-HSCR). The preoperative diagnostic sensitivity of SCGN was 100%, with a specificity of 98.72%. *F*, biopsy samples from the control and HSCR groups were analyzed for SCGN; *blue arrows* show the heterostained cells appearing for both proteins (goblet cells in the mucosal crypts of endodermis), which can be used as inner control. LEF-1, lymphoid enhancer-binding factor 1; NCAM, neural cell adhesion molecule; SCGN, secretagogin.

To elucidate the clinical utility of SCGN in diagnosing HSCR, we conducted immunohistochemical staining of colon tissues from patients with and without HSCR. We further examined the expression of the SCGN, LEF-1, and NCAM1 axis in clinical specimens using WB assays (Fig. 7*A*). All three proteins exhibited differential downregulation in HSCR. In serial section specimens labeled by immunohistochemical staining (Fig. 7*B*), we observed that SCGN, LEF-1, and NCAM1 were either negative or weakly positive in the myenteric plexus region of HSCR colonic tissues, whereas they were strongly positive in the control group. This finding indicates a consistent expression pattern of the SCGN–LEF-1–NCAM1 axis in clinical tissue specimens. Additionally, based on the immunohistochemical staining results, we identified the potential of SCGN as a pathological marker for HSCR. Therefore, we observed positive immunoexpression of SCGN in the submucosal and intermuscular layers of non-HSCR, while it was negative in HSCR (Fig. 7*C*), suggesting that SCGN mucosal biopsy may be a valuable diagnostic tool for HSCR. To validate this hypothesis, we analyzed data from 130 children with constipation who underwent mucosal biopsy and surgical treatment at our hospital. The cohort included 92 males and 38 females with an average age of 15.7 months. The pathological types of these cases were determined by H&E stain. Among the 130 cases, 52 were diagnosed with HSCR and 78 with non-HSCR. Comparing the SCGN staining results of preoperative mucosal biopsies with the postoperative pathological diagnoses, we found that SCGN exhibited a sensitivity of 100% and a specificity of 98.72% for diagnosing HSCR (Fig. 7, *D* and *E*). Moreover, positive staining of SCGN in goblet cells can serve as an internal reference to enhance the reliability of this staining method (Fig. 7*F*). In conclusion, our findings demonstrate that SCGN has high sensitivity and specificity for diagnosing HSCR and holds promising potential for clinical application.

## Discussion

This study demonstrated that SCGN expression was downregulated in HSCR lesion colon tissues and that SCGN deletion inhibited cell migration. These findings suggest a role for SCGN in enteric neural crest cell development and the pathogenesis of HSCR. The knockdown of SCGN suppressed NCAM1 transcriptional activation by downregulating the transcription factor LEF-1, which subsequently attenuated the cells' ability to migrate. Notably, SCGN mucosal biopsy exhibits high sensitivity and specificity for diagnosing HSCR, indicating its potential as a novel diagnostic marker.

The normal development of the ENS is the result of a complex interplay between enteric neural crest cells and the extracellular environment (14). Comparing the lesion colon tissues from HSCR with the normal colon may provide insights into the factors influencing the development of ENS. Herein, we conducted protein profiling to identify differentially expressed proteins between HSCR and normal colonic tissues obtained from trauma patients. Our analysis revealed significantly lower expression levels of SCGN in HSCR than the control group. Further experiments confirmed this finding and suggested a close association between SCGN expression and localization and the presence of enteric ganglion cells. Previous studies have reported that SCGN is a calcium-modulated binding protein exclusively expressed in the neuroendocrine system (15, 16). The research reports that glutamatergic neurons in the ventromedial indusium griseum sequentially express the transcription factors Pou3f3/Brn1 and SCGN during differentiation. Pou3f3/Brn1, a key regulator of cortical pyramidal neuron differentiation, likely controls the temporal expression of SCGN by initiating its transcriptional program (17). This regulatory pathway may contribute to the observed downregulation of SCGN. Beyond its endocrine functions, SCGN is implicated in downstream neural development processes. Previous studies have reported that SCGN can mediate MMP-2 cytosolization and activation via annexin V, leading to extracellular matrix restructuring and promoting the process of central nervous system rostral migratory stream (11). In this study, we investigated the biological function of SCGN using SH-SY5Y and SK-N-BE(2) cells, which possess the functional characteristics of enteric neural crest cells with different degrees of differentiation. Our results demonstrated that SCGN significantly enhanced the migration of neuronal cells without affecting their proliferation ability. Through zebrafish model studies, we further validated the functional role of SCGN in regulating nervous system development. These findings suggest that low SCGN expression may contribute to the pathogenesis of HSCR by inhibiting the migration of intestinal neural crest cells.

NCAM1, also known as CD56, is a protein expressed on the surface of neurons, astrocytes, and immune cells (18, 19). Its biological functions include cell adhesion and nerve growth support (20, 21). Knockdown of NCAM1 during embryonic development leads to impaired mouse central nervous system development (22). NCAM1 has been implicated in various human psychiatric disorders, including bipolar affective disorder, schizophrenia, and autism spectrum disorders (23, 24). Previous studies have shown that NCAM1 is expressed at low levels in the aganglionic colon tissues, suggesting a potential role in the pathogenesis of this condition (25). Through protein profiling and database analysis, NCAM1 was identified as a candidate downstream molecule of SCGN. The positive regulatory effect of SCGN on NCAM1 was confirmed by qRT-PCR and WB. Notably, overexpression of NCAM1 promoted cell migration and could counteract the decreased migratory ability of neuronal cells caused by SCGN silencing, suggesting that NCAM1 is a key substrate for the promigratory role of SCGN. To investigate the mechanism by which SCGN regulates NCAM1 expression, we focused on LEF-1, a transcription factor known to upregulate NCAM1 promoter activity (13, 26). Using the JASPAR database, we predicted the binding site of LEF-1 to NCAM1 and confirmed its direct binding to the NCAM1 promoter region, leading to transcriptional activation, as demonstrated by luciferase assays. Additionally, we observed that LEF-1 expression was downregulated upon SCGN silencing. Exogenous overexpression of LEF-1 could counteract the downregulation of NCAM1 expression and the inhibition of cell migration caused by SCGN silencing. Furthermore, in clinical samples, we observed consistent differential expression patterns of SCGN, LEF-1, and NCAM1, which provides indirect evidence for the existence of this signaling pathway in the human ENS. However, SCGN is not a transcriptional regulator, suggesting that its modulation of LEF-1 is likely involves intermediate mechanisms. Previous studies have reported that LEF-1 is positively regulated by activated NF-ĸB, while SCGN can upregulate TNFα and subsequently activate the NF-ĸB pathway. Therefore, we hypothesize that the TNFα/NF-ĸB signaling axis may serve as a key mechanism through which SCGN regulates LEF-1 expression. Nevertheless, this conclusion requires further experimental validation. In summary, SCGN directly induces NCAM1 transcriptional activation through the upregulation of the transcription factor LEF-1, which in turn promotes cell migration.

Beyond the molecular mechanisms, a more intriguing finding of this study is the potential clinical significance of SCGN. Early symptoms are atypical in some newborns with HSCR, leading to misdiagnosis and underdiagnosis due to physiological peculiarities (27). Research has shown that delayed diagnosis of HSCR increases the risk of preoperative complications such as Hirschsprung-associated enterocolitis and perforation, which can be life-threatening (28). Consequently, early definitive diagnosis and precise management are crucial. Currently, the most important preoperative examination modalities for HSCR include barium enema, high-resolution rectal anorectal manometry, and rectal mucosal biopsy (29). Among these, mucosal biopsies offer the highest diagnostic accuracy. However, existing biopsy markers have limitations. Calretinin is the most commonly used biomarker for HSCR currently, used to label enteric ganglion cells (2, 30). However, mast cell staining has been reported with calretinin, and mast cells and enteric ganglion cells are located in the submucosa, making it difficult to distinguish between them and resulting in a false-positive rate of 3% to 16% (31, 32). Therefore, identifying novel biopsy markers is essential for improving HSCR diagnostic accuracy. Our immunohistochemical results demonstrate that SCGN accurately labels intermuscular and submucosal ganglion cells, making it a potential mucosal biopsy marker. To assess the diagnostic efficacy of SCGN, we collected 130 cases of children with constipation who underwent mucosal biopsy and surgical treatment at our institution. Comparing SCGN staining results of preoperative mucosal biopsy with postoperative pathology results, we found that all children with HSCR had a negative SCGN mucosal biopsy, while 98.72% of children with non-HSCR constipation had a positive SCGN result. This indicates that SCGN mucosal biopsy has high potential for diagnosing HSCR with both sensitivity and specificity. In addition to the ganglion cell, a small number of goblet cells can also be stained, but fortunately the goblet cells are located within the mucosal crypts on the mucosal surface. Microscopically, the goblet cells are surrounded by intestinal mucosal epithelial cells in a circle and ganglion cells are outside the circle (33). The distinction between ganglion cells and goblet cells can be readily made on the basis of their respective locations, as shown in Figure 6. This approach has the advantage of reducing the false-positive rate. It is noteworthy that the staining in mucosal crypts could serve as an internal positive reference, enhancing the consistency of the pathologist's assessment. Therefore, SCGN is expected to be a promising diagnostic marker for HSCR in clinical applications. However, several challenges remain in advancing its clinical translation, including inconsistent antibody specificity and subjective interpretation of staining results. Key solutions involve screening highly specific antibodies, developing digital interpretation tools, and establishing standardized diagnostic protocols. Furthermore, multicenter clinical studies should be actively conducted to rigorously validate the diagnostic efficacy of SCGN as a biomarker and evaluate its practical benefits in reducing diagnostic delays and secondary surgery rates within real-world clinical workflows.

In conclusion, our study demonstrates that SCGN promotes the migration of intestinal neural crest cells by upregulating the LEF-1/NCAM1 axis. This finding contributes to our understanding of the pathogenesis of HSCR and provides a theoretical foundation for SCGN as a novel diagnostic marker for HSCR.

## Limitations

This study identified SCGN as a promising diagnostic biomarker for HSCR through proteomic profiling. However, several limitations should be noted. First, our biomarker screening was confined to top-ranked differentially expressed proteins, and we did not perform immunohistochemical validation of other potential protein markers. Future studies will further explore additional candidate markers. Second, the diagnostic efficacy of SCGN was not systematically compared with established biomarkers (*e.g.*, retinal-binding protein, peripherin), and the clinical utility of combining SCGN with existing markers warrants further investigation. Regarding mechanistic insights, while we validated SCGN's biological function in animal models and demonstrated the existence of the SCGN/LEF-1/NCAM1 axis in human specimens, more detailed investigations are needed to elucidate this signaling pathway, particularly the upstream regulators of SCGN. Addressing these research gaps will provide a more comprehensive understanding of HSCR pathogenesis and potentially reveal novel therapeutic strategies.

## Data Availability

All data are available under reasonable request. All mass spectrometry raw data and output files were uploaded to iProX (PXD058158) and all can be downloaded *via* ProteomeXchange with their respective identifier.

## Ethical Approval

The studies involving animal were reviewed and approved by Institutional Animal Care and Use Committee, Tongji Medical College, Huazhong University of Science and Technology, with the approval in Supplemental materials.

## Conflict of Interest

The authors declare no competing interests.

## Supplemental Data

Supplemental Table S5

Ethics approval of Clinical Trial

Ethics approval of Animal Experiment

Annotated spectra

## References

[1] Heuckeroth R.O. (2018). Hirschsprung disease - integrating basic science and clinical medicine to improve outcomes. Nat. Rev. Gastroenterol. Hepatol..

[2] Montalva L., Cheng L.S., Kapur R., Langer J.C., Berrebi D., Kyrklund K. (2023). Hirschsprung disease. Nat. Rev. Dis. Primers.

[3] Hagens J., Reinshagen K., Tomuschat C. (2022). Prevalence of Hirschsprung-associated enterocolitis in patients with Hirschsprung disease. Pediatr. Surg. Int..

[4] Dershowitz L.B., Kaltschmidt J.A. (2024). Enteric nervous system striped patterning and disease: unexplored pathophysiology. Cell Mol. Gastroenterol. Hepatol..

[5] Torroglosa A., Villalba-Benito L., Luzon-Toro B., Fernandez R.M., Antinolo G., Borrego S. (2019). Epigenetic mechanisms in Hirschsprung disease. Int. J. Mol. Sci..

[6] Qin J., Liu Q., Liu Z., Pan Y.Z., Sifuentes-Dominguez L., Stepien K.P. (2020). Structural and mechanistic insights into secretagogin-mediated exocytosis. Proc. Natl. Acad. Sci. U. S. A..

[7] Maj M., Wagner L., Tretter V. (2019). 20 Years of secretagogin: exocytosis and beyond. Front. Mol. Neurosci..

[8] Chidananda A.H., Sharma A.K., Khandelwal R., Sharma Y. (2019). Secretagogin binding prevents alpha-synuclein fibrillation. Biochemistry.

[9] Liu Z., Tan S., Zhou L., Chen L., Liu M., Wang W. (2023). SCGN deficiency is a risk factor for autism spectrum disorder. Signal. Transduct. Target. Ther..

[10] Wang Q.W., Qin J., Chen Y.F., Tu Y., Xing Y.Y., Wang Y. (2023). 16p11.2 CNV gene Doc2alpha functions in neurodevelopment and social behaviors through interaction with Secretagogin. Cell Rep..

[11] Hanics J., Szodorai E., Tortoriello G., Malenczyk K., Keimpema E., Lubec G. (2017). Secretagogin-dependent matrix metalloprotease-2 release from neurons regulates neuroblast migration. Proc. Natl. Acad. Sci. U. S. A..

[12] Verpoort B., de Wit J. (2024). Cell adhesion molecule signaling at the synapse: beyond the scaffold. Cold Spring Harb. Perspect. Biol..

[13] Boras K., Hamel P.A. (2002). Alx4 binding to LEF-1 regulates N-CAM promoter activity. J. Biol. Chem..

[14] Ji Y., Tam P.K., Tang C.S. (2021). Roles of enteric neural stem cell niche and enteric nervous system development in Hirschsprung disease. Int. J. Mol. Sci..

[15] Sifuentes-Dominguez L.F., Li H., Llano E., Liu Z., Singla A., Patel A.S. (2019). SCGN deficiency results in colitis susceptibility. Elife.

[16] Sharma A.K., Khandelwal R., Sharma Y. (2019). Veiled potential of secretagogin in diabetes: correlation or coincidence?. Trends Endocrinol. Metab..

[17] Fuzik J., Rehman S., Girach F., Miklosi A.G., Korchynska S., Arque G. (2019). Brain-wide genetic mapping identifies the indusium griseum as a prenatal target of pharmacologically unrelated psychostimulants. Proc. Natl. Acad. Sci. U. S. A..

[18] Van Acker H.H., Capsomidis A., Smits E.L., Van Tendeloo V.F. (2017). CD56 in the immune system: more than a marker for cytotoxicity?. Front. Immunol..

[19] Gallo P.N., Mihelc E., Eisert R., Bradshaw G.A., Dimek F., Leffler A. (2024). The dynamic TRPV2 ion channel proximity proteome reveals functional links of calcium flux with cellular adhesion factors NCAM and L1CAM in neurite outgrowth. Cell Calcium.

[20] Sun Y., Liu Q., Qin Y., Xu Y., Zhao J., Xie Y. (2024). Exosomes derived from CD271+CD56+ bone marrow mesenchymal stem cell subpopoulation identified by single-cell RNA sequencing promote axon regeneration after spinal cord injury. Theranostics.

[21] Sun J.H., Huang M., Fang Z., Li T.X., Wu T.T., Chen Y. (2022). Nerve bundle formation during the promotion of peripheral nerve regeneration: collagen VI-neural cell adhesion molecule 1 interaction. Neural Regen. Res..

[22] Rai S., Leydier L., Sharma S., Katwala J., Sahu A. (2023). A quest for genetic causes underlying signaling pathways associated with neural tube defects. Front. Pediatr..

[23] Eve M., Gandawijaya J., Yang L., Oguro-Ando A. (2022). Neuronal cell adhesion molecules may mediate neuroinflammation in autism spectrum disorder. Front. Psychiatry.

[24] Khlidj Y., Haireche M.A. (2023). Schizophrenia as autoimmune disease: involvement of Anti-NCAM antibodies. J. Psychiatr. Res..

[25] Yoshimaru K., Taguchi T., Obata S., Takemoto J., Takahashi Y., Iwanaka T. (2017). Immunostaining for Hu C/D and CD56 is useful for a definitive histopathological diagnosis of congenital and acquired isolated hypoganglionosis. Virchows Arch..

[26] Morgan R.G., Ridsdale J., Payne M., Heesom K.J., Wilson M.C., Davidson A. (2019). LEF-1 drives aberrant beta-catenin nuclear localization in myeloid leukemia cells. Haematologica.

[27] Ambartsumyan L., Smith C., Kapur R.P. (2020). Diagnosis of Hirschsprung disease. Pediatr. Dev. Pathol..

[28] Zhang Y., Xiang X., Li X., Feng W., Guo Z. (2024). Early intervention in Hirschsprung's disease: effects on enterocolitis and surgical outcomes. BMC Pediatr..

[29] Gong Y.-Y., Lv J.-J., Yang T., Huang X.-Z., Zhang L., Wu J.-H. (2022). Systematic appraisal of the guidelines for the diagnosis and treatment of Hirschsprung's disease. Pediatr. Surg. Int..

[30] Beltman L., Windster J.D., Roelofs J., van der Voorn J.P., Derikx J.P.M., Bakx R. (2022). Diagnostic accuracy of calretinin and acetylcholinesterase staining of rectal suction biopsies in Hirschsprung disease examined by unexperienced pathologists. Virchows Arch..

[31] Alexandrescu S., Rosenberg H., Tatevian N. (2013). Role of calretinin immunohistochemical stain in evaluation of Hirschsprung disease: an institutional experience. Int. J. Clin. Exp. Pathol..

[32] Cinel L., Ceyran B., Gucluer B. (2015). Calretinin immunohistochemistry for the diagnosis of Hirschprung disease in rectal biopsies. Pathol. Res. Pract..

[33] Schumacher M.A. (2023). The emerging roles of deep crypt secretory cells in colonic physiology. Am. J. Physiol. Gastrointest. Liver Physiol..

