# Supplementary material for: Secretagogin Downregulation Impairs Nerve Cell Migration in Hirschsprung Disease via Inhibition of the LEF-1/NCAM1 Axis
Source: Mol Cell Proteomics. 2025 Jul 11;24(8):101032. doi: 10.1016/j.mcpro.2025.101032 (PMC12359226; doi:10.1016/j.mcpro.2025.101032)
Supplement: Supplementary Material [file mmc1.docx]

| **Genes** | **Sequences (5'--3')** |
| --- | --- |
| Scramble si-RNA | UUCUCCGAACGUGUCACGU |
| Si-SCGN 1 | GUUCGCAUCUUACCACAUA |
| Si-SCGN 2 | UCAUAAACUGCUGUUUCACCU |
| Si-LEF-1 | AUUAUGUACCCGGAAUAACUC |

**Table S1.** Sequences of Si-RNA used for functional experiment in vitro.

| **Genes** | **Sequences (5'--3')** |
| --- | --- |
| SCGN | F: CCCAGAAGTGGATGGATTTG |
|  | R: GTTGGGGATCAGGGGTTTAT |
| NCAM1 | F: TTCCTGTGTCAAGTGGCAGG |
|  | R: TAAACTCCTGTGGGGTTGGC |
| LEF-1 | F: AGAACACCCCGATGACGGA |
|  | R: GGCATCATTATGTACCCGGAAT |
| CADM3 | F: CAAGTGCCAAGTGAAAGATCACG |
|  | R: CCCAAAGTAGAGAGTCTGCTGAG |
| CNTN1 | F: CAGCCCTTTCCCGGTTTACAA |
|  | R: TGCTTCTGACCATCCCGTAGT |
| CADM1 | F: GACGTGACAGTGATCGAGGG |
|  | R: GGGATCGGTATAGAGCTGGCA |
| L1CAM | F: TGTCATCACGGAACAGTCTCC |
|  | R: CTGGCAAAGCAGCGGTAGAT |
| ALCAM | F: TCCTGCCGTCTGCTCTTCT |
|  | R: TTCTGAGGTACGTCAAGTCGG |
| GAPDH | F: AGAAGGCTGGGGCTCATTTG |
|  | R: AGGGGCCATCCACAGTCTTC |

F, forward primer; R, reverse primer.

**Table S2.** Sequences of primers used for Real-time quantitative PCR.

| **Genes** | **Sequences (5'--3')** |
| --- | --- |
| SCGN-MO1 | GGTTGGCAAAAGCACTGTCCATGAT |
| SCGN-MO2 | GTGGTTTATGTTTGATACCTTTGGC |
| Control | CCTCTTACCTCAGTTACAATTTATA |

**Table S3.** Sequences of MO and control used for functional experiment in zebrafish.

| Items | Value |
| --- | --- |
| Protein Database | Uniprot_HomoSapiens_20386_20180905 |
| Enzyme | Trypsin |
| Max Missed Cleavages | 2 |
| Instrument | ESI-TRAP |
| Precursor Mass Tolerance | ± 10 ppm |
| Fragment Mass Tolerance | 0.05Da |
| Use Average Precursor Mass | False |
| Modification Groups From Quan Method | TMT 10plex |
| Dynamic modifications | Oxidation (M) |
|  | Acetyl (Protein N-term) |
| Static modifications | Carbamidomethyl (C) |
| Database pattern | decoy |
| Peptide FDR | ≤0.01 |

**Table S6.** Additional search parameters and acceptance criteria for peptide/protein identification.

| Items | Value |
| --- | --- |
| Accession | Protein numbering in the FASTAddatabase |
| Gene Name | The gene name annotated in the Fasthead column will not be displayed if the annotation information in the database is incomplete or there is no gene name. |
| Description | Functional descriptions of proteins in databases based on protein sequences. |
| Coverage | The percentage of the protein sequence covered by identified peptides |
| # Peptides | The number of distinct peptide sequences in the protein group |
| # PSMs | The total number of identified peptide sequences for the protein, including those redundantly identified. |
| # Unique Peptides | The total number of identified peptide sequences for the protein, including those redundantly identified. |
| # AAs | Total number of amino acids in a protein |
|  |  |
| (Continued table) |  |
| Items | Value |
| MW [kDa]  calc. pI | Theoretical molecular weight of a protein. The molecular weight is calculated by the software based on protein sequences in a database. If the protein sequence used for the calculation is not a complete full-length sequence, such as a protein sequence translated from the transcriptome, the calculated molecular weight will be less than the molecular weight of the entire protein. |
|  | Theoretical isoelectric point of protein |
| Abundances T1 | Relative expression amount of protein in HSCR group 1 |
| Abundances T2 | Relative expression amount of protein in HSCR group 2 |
| Abundances T3 | Relative expression amount of protein in HSCR group 3 |

**Table S7.** Table legend for Table S4.

| Items | Value |
| --- | --- |
| Sequence | Describe the amino acid composition of the peptide segment |
| Modifications | Describe the modified amino acid, position and modification method. For example: C(Carbamidomethyl): cysteine modification; M(Oxidation): methionine oxidation; S/T/Y (Phosphorylation): serine/threonine/tyrosine phosphorylation |
| Qvality PEP | Local FDR. The posterior error probability (PEP) is the probability that the observed PSM is incorrect. This value essentially operates as a p-value, where smaller is better. For example, if the PEP associated with (EAMRPK, s) is 5 percent, there is a 95 percent chance that the EAMRPK peptide was in the mass spectrometer when spectrum s was generated. The FDR measures the error rate associated with a collection of PSMs, and the PEP measures the probability of error for a single PSM. |
|  |  |
| (Continued table) |  |
| Items | Value |
| Qvality q-value | Global FDR. A q-value is the minimal false discovery rate at which the identification is considered correct. q-values are estimated using the distribution of scores from the decoy database search. A q-value of 0.01 for the EAMRQPK peptide matching spectrum, s, means that if you try all possible FDR thresholds, 1 percent is the minimal FDR threshold at which the PSM of EAMRQPK to s appears in the output list. Although the q-value is associated with a single PSM, it also depends on the data set that the PSM occurs in. |
| # PSMs | peptide spectrum matches, The total number of identified peptide sequences for the protein, including those redundantly identified. |
| Master Protein Accessions | Protein numbering in the FASTAddatabase |
| # Missed Cleavages | Identify the number of trypsin missed cleavage sites (i.e. K and R) in the peptide sequence |
|  |  |
| (Continued table) |  |
| Items | Value |
| Theo. MH+ [Da] | Theoretical molecular weight of a peptide with one charge (protonated) |
| Abundances T1  Abundances T2 | Relative abundance of peptide segments in HSCR group 1 |
|  | Relative abundance of peptide segments in HSCR group 2 |
| Abundances T3 | Relative abundance of peptide segments in HSCR group 3 |
| Abundances I1 | Relative abundance of peptide segments in control group 1 |
| Abundances I2 | Relative abundance of peptide segments in control group 2 |

**Table S8.** Table legend for Table S5.
