# Supplementary material for: Secretagogin Downregulation Impairs Nerve Cell Migration in Hirschsprung Disease via Inhibition of the LEF-1/NCAM1 Axis
Source: Mol Cell Proteomics. 2025 Jul 11;24(8):101032. doi: 10.1016/j.mcpro.2025.101032 (PMC12359226; doi:10.1016/j.mcpro.2025.101032)
Supplement: Ethics approval of Clinical Trial [file mmc4.pdf]

华中科技大学同济医学院医学伦理委员会

伦理审查批件

[2016]伦审字（S180）号　　　　审查日期：2017年06月21日

研究项目名称	小儿腔镜诊断治疗消化道畸形技术规范、标准及新技术评价研究			
项目来源	类别	☐企业　☐学术团体　☐基金会　☐国际组织　☐院、校课题　☐研究生课题<br>☒其他：政府		
	名称	卫计委公益性行业科研专项		
申请单位/科室	华中科技大学同济医学院附属协会医院/小儿外科		项目负责人/职称	汤绍涛/教授
合同研究组织	NA			
审查文件	1. 复审申请表<br>2. 研究方案<br>3. 知情同意书			
审查方式	■快速审查　审查时间：2016年07月13日<br>■快速审查　审查时间：2017年05月31日			
审查委员	田德英　　李丽平			
审查意见	1. 经本伦理委员会审查，同意按所批准的研究方案进行该临床研究。<br>2. 伦理委员会对该研究实施过程进行持续审查，审查频度为研究批准之日（2017日06月21日）起：<br>☐3个月　☐6个月　■1年<br>3. 伦理委员会有权根据研究进展情况改变持续审查频度。			

本伦理委员会的人员组成和工作程序遵循ICH-GCP、中国GCP及相关法律法规。

注意：（请仔细阅读）

1. 须按照本伦理委员会已批准的方案开展临床研究，须符合GCP和《赫尔辛基宣言》的原则，保护受试者的健康和权益。
2. 对已批准的临床研究方案、知情同意书等相关文件所作的任何修改，须及时报告本伦理委员会，经重新审查同意后方可实施。
3. 本中心发生的严重不良事件或非预期不良事件，须及时向本伦理委员会作出书面通报，伦理委员会有权根据对其评估作出新的决定。
4. 根据伦理委员会对持续审查频度的意见，无论试验开始与否，请在持续审查日到期前1个月提出再次审查的申请。
5. 暂停/提前终止临床研究，须及时向伦理委员会提交暂停/终止试验报告。
6. 如有不依从/违背方案情况须及时向伦理委员会书面报告。
7. 临床试验结束后，须提交结题报告供伦理委员会审查。
8. 凡涉及人类遗传资源出口或者按照国家规定必须经有关部门专项审批的研究内容，均须遵循国家相关法规及规定向有关部门申报并获批准。
9. 本伦理批件自批准之日起有效期1年，逾期未实施本批件自行作废。

华中科技大学同济医学院医学伦理委员会（盖章）：

主任/副主任委员签名：[illegible]

日期：2017.6.21.

地址：武汉市航空路13号　邮编：430030　电话&传真：027-83691785　Email：tongjilunli@163.com
