# Supplementary material for: Secretagogin Downregulation Impairs Nerve Cell Migration in Hirschsprung Disease via Inhibition of the LEF-1/NCAM1 Axis
Source: Mol Cell Proteomics. 2025 Jul 11;24(8):101032. doi: 10.1016/j.mcpro.2025.101032 (PMC12359226; doi:10.1016/j.mcpro.2025.101032)
Supplement: Ethics approval of Animal Experiment [file mmc5.pdf]

766

华中科技大学实验动物伦理委员会动物伦理备案审批报告

项目名称	SCGN 低表达介导胞膜 L1CAM 下调抑制肠神经嵴细胞迁移在先天性巨结肠发病中的机制研究		
申请单位	华中科技大学		
项目负责人	周韵	职称	无
报送资料	课题研究方案	有☑ 无☐	
	研究人员名单	有☑ 无☐	
审查	研究者资格	符合要求☑ 不符合要求☐	
	课题研究方案	适当☑ 不适当☐	
有效期	2025 年 01 月 01 日至 2027 年 12 月 31 日		

审评意见：

本伦理委员会审阅并讨论了上述相关资料，该课题研究符合《湖北省实验动物管理条例》和《华中科技大学实验动物伦理委员会章程》，经伦理委员会审核，同意该课题实施。

华中科技大学实验动物伦理委员会

批准日期： 2024 年 3 月 6 日

华中科技大学 实验动物伦理委员会

仅用于国家自然基金实验动物福利伦理备案申报使用
